# Cytokine Profile in Chronic Periodontitis Patients with and without Obesity: A Systematic Review and Meta-Analysis

**DOI:** 10.1155/2016/4801418

**Published:** 2016-09-26

**Authors:** Zohaib Akram, Tariq Abduljabbar, Mohamed Ibrahim Abu Hassan, Fawad Javed, Fahim Vohra

**Affiliations:** ^1^Department of Periodontology, Faculty of Dentistry, University of Malaya, Kuala Lumpur, Malaysia; ^2^Department of Periodontology, Faculty of Dentistry, Ziauddin University, Karachi, Pakistan; ^3^Department of Prosthetic Dental Sciences, College of Dentistry, King Saud University, Riyadh, Saudi Arabia; ^4^Department of Restorative Dentistry, Faculty of Dentistry, MARA University of Technology, Selangor, Malaysia; ^5^Department of General Dentistry, Eastman Institute for Oral Health, University of Rochester, Rochester, NY 14620, USA

## Abstract

To investigate the cytokine profile as biomarkers in the gingival crevicular fluid (GCF) of chronic periodontitis (CP) patients with and without obesity, MEDLINE/PubMed, EMBASE, ScienceDirect, and SCOPUS databases were combined with handsearching of articles published from 1977 up to May 2016 using relevant MeSH terms. Meta-analyses were conducted separately for each of the cytokines: resistin, adiponectin, TNF-*α*, leptin, IL-6, IL-8, and IL-1*β*. Forest plots were produced reporting standardized mean difference of outcomes and 95% confidence intervals. Eleven studies were included. Three studies showed comparable levels of leptin among obese and nonobese patients with CP. Four studies reported comparable levels of interleukin- (IL-) 6 and resistin whereas five studies reported comparable levels of adiponectin. Two studies reported similar levels of CRP in patients with periodontitis with and without obesity. One study showed higher levels of tumor necrosis factor-alpha in obese patients with CP. One study showed higher levels of IL-1*β* and IL-8 in obese patients with CP. The level of localized periodontal inflammation may have a greater influence on the GCF proinflammatory biomarker levels as compared to systemic obesity. Whether patients having chronic periodontitis with obesity have elevated proinflammatory GCF biomarkers levels compared to nonobese individuals remains debatable.

## 1. Introduction

Obesity is described as a condition associated with expansion in adipocytes and increased infiltration of macrophage cells in the adipose tissues, defining the inflammatory state [1, 2]. Substantial evidence in the form of in vivo and in vitro studies has demonstrated a strong association between chronic periodontitis (CP) and obesity [3–6]. The underlying mechanisms of periodontitis in obesity are not well understood; however, it is suggested that the increased levels of proinflammatory cytokines as biomarkers (such as interleukin- (IL-) 6, IL-1*β*) in the gingival crevicular fluids (GCF) of obese individuals are associated with poor periodontal health [7].

Cytokines are low molecular weight water-soluble glycoprotein biomarkers secreted by hematopoietic and nonhematopoietic cells in response to infection. Inflammatory biomarkers which are induced during inflammatory responses have been associated with the onset or progression of tissue insult [8, 9]. It is suggested that proinflammatory biomarkers show pleiotropic effect and can target specific cells by controlling activation of cells, cell proliferation, and function in the periodontium [10, 11]. As a result, raised proinflammatory biomarkers levels such as tumor necrosis factor-alpha (TNF-*α*), IL-1*β*, IL-6, and IL-8 cause periodontal tissue destruction [12]. The reason for studying these proinflammatory cytokines in obesity is to validate the association of the same cytokines which are actively involved in jeopardizing periodontal tissues by mediating alveolar bone resorption and collagen destruction [12].

Obese patients are associated with a state of elevated systemic inflammatory burden due to increased serum proinflammatory cytokine levels [13, 14]. In addition, studies have shown increased susceptibility of inflammatory periodontal tissue destruction in overweight or obese patients as compared to healthy individuals [15]. Moreover, inflammatory cytokine mediated mechanisms are implicated in periodontal inflammatory conditions [11]. In this context, it may be hypothesized that the GCF cytokine profile in obese patients with CP should be elevated as compared to nonobese individuals with periodontal disease. In the study by Modéer et al. [16] GCF IL-1*β* and IL-8 levels among obese patient with CP were significantly higher as compared to nonobese CP patients. In contrast, Duzagac et al. [17] showed comparable GCF cytokine profile among obese and nonobese patients with CP. There appears to be a controversy with regard to GCF cytokine profile in chronic periodontitis patients with and without obesity. Therefore, the aim of this study was to systematically review the GCF cytokine profile in CP patients with and without obesity.

## 2. Methods

### 2.1. Protocol and Registration

This review was registered at the National Institute for Health Research PROSPERO, International Prospective Register of Systematic Reviews (http://www.crd.york.ac.uk/PROSPERO/, registration number CRD42015029928). Based on the Preferred Reporting Items for Systematic Review and Meta-Analysis (PRISMA) guidelines [18], a specific question was constructed. The addressed focused question was “Does the GCF cytokine profile of obese and nonobese patients with chronic periodontitis differ?”

### 2.2. Selection Criteria

The following eligibility criteria were entailed:

- clinical trials (baseline data), cross-sectional and observational (prospective; baseline data) studies in humans (adults and adolescents only) using any type of assay method;
- cytokine profile in the GCF of patients with chronic periodontitis with and without obesity.

In vitro studies; animal studies; studies providing analyses of cytokines in fluids other than GCF; studies that reported cytokine profile among obese only with no normal weight controls; letters to the editor; and review papers and unpublished articles were excluded.

### 2.3. Search Strategy

Two reviewers (Z. A. and T. A.) searched the following electronic databases: (1) MEDLINE, (2) PubMed, (3) EMBASE, (4) ISI Web of Knowledge, (5) ScienceDirect, and (6) SCOPUS from 1977 up to May 2016 for appropriate articles addressing the focused question. A structured approach to literature search algorithm was used to explore databases, in which Boolean operators and the asterisk were used as truncation (“Periodontitis” [MeSH terms] OR “Chronic Periodontitis” [MeSH terms] OR “Periodontal Diseases” [MeSH terms] AND (“Cytokines” [MeSH terms] OR “Adipokines” [MeSH terms] OR “Adipocytokines” OR “Biomarkers” OR “Pro-inflammatory”) AND (“Gingival Crevicular Fluid” OR “Crevicular Fluid” [MeSH terms] OR “Sulcular Fluid”) AND (“Obesity” [MeSH terms] OR “Obese” OR “Body Mass Index [MeSH terms]” OR “Adiposity” OR “Body Weight” OR “Waist circumference” OR “Waist-Hip Ratio”).

### 2.4. Screening and Selection

**Figure 1:**
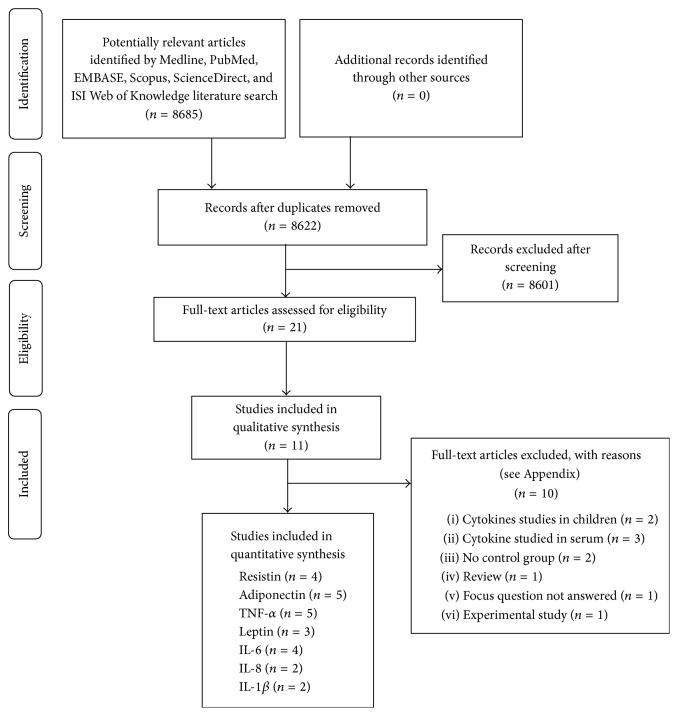
PRISMA flow diagram for studies retrieved through the searching and selection process.

Two reviewers (Z. A. and F. V.) independently screened titles and abstracts for eligible papers. If information relevant to the eligibility criteria was not available in the abstract or if the title was relevant but the abstract was not available, the paper was selected for full reading of the text. Next, full-text papers that fulfilled the eligibility criteria were identified and included in the review. Following that, reference lists of original studies were handsearched to identify articles that could have been missed during the electronic search. Handsearching of the following journals was performed:* Journal of Clinical Periodontology, Journal of Periodontology, Journal of Periodontal Research, Journal of Dental Research, Journal of Periodontology and Implant Dentistry, Clinical Oral Investigations, Brazilian Dental Journal, Saudi Medical Journal, Journal of Indian Society of Periodontology, Cytokine, Journal of Investigative and Clinical Dentistry, Disease Markers,* and* International Journal of Pediatric Obesity.* Papers that fulfilled all of the selection criteria were processed for data extraction.  Figure 1 describes the screening process according to PRISMA guidelines for flow diagram [18].

### 2.5. Data Extraction and Quality Assessment

Two reviewers (Z. A. and F. V.) undertook this independently. The information from the accepted studies was tabulated according to the study designs, subject characteristics, sample characteristics, cytokines investigated, and main outcomes. Data collected were based on the focused question outlined for the present systematic review. Baseline data that compared the levels of cytokines among obese and nonobese patients in prospective studies were also included in the review. The reviewers crosschecked all extracted data. Any disagreement was resolved by discussion until consensus was reached. The quality of the included studies was assessed using the Newcastle-Ottawa Quality Assessment scale for Observational Studies [27] (Table 3), a validated scale for evaluating the quality of observational and nonrandomized studies. This scale uses a star system to evaluate the studies on three broad perspectives: the selection of the study groups; the comparability of the groups; and the ascertainment of either the exposure or outcome of interest for included studies respectively.

### 2.6. Statistical Analyses

Meta-analyses were conducted separately for each of the cytokines: resistin, adiponectin, TNF-*α*, leptin, IL-6, IL-8, and IL-1*β*. Heterogeneity among the included studies for each cytokine outcome was assessed using the *Q*-statistic and *I* ^2^ statistic [28]. Outcome measures for each inflammatory mediator were combined with a random-effects model utilizing the DerSimonian-Laird method due to its robustness in comparison to fixed-effects models in the case of small sample sizes [29]. Forest plots were produced reporting standardized mean difference (SMD) of outcomes and 95% confidence intervals (CI). The pooled effect was considered significant if *P* value was <0.05. All above statistical analyses were carried out by a specialized statistical software (MedCalc Software—B-8400 Ostend v. 15.11.04, Belgium).

## 3. Results

### 3.1. Study Selection

From an original yield of 8685 articles, a total of 21 studies were accepted for full-text review. After full-text review, 10 more studies were excluded that did not fulfil the inclusion criteria (see Appendix with reasons for exclusion). A total of 11 studies [6, 16, 17, 19–26] were included in the present systematic review. All studies were performed at either university clinics [6, 16, 17, 19–23, 25, 26] or health care centres [24]. The kappa value for interreviewer agreement was (95% confidence interval): 0.82 (0.78–0.87).

### 3.2. Qualitative Results of Studies

Eleven studies [6, 16, 17, 19–26] included in the present review enlisted eight cross-sectional [6, 16, 20, 21, 23–26] and three prospective intervention studies [17, 19, 22] (Table 1). The total number of participants in the included studies ranged between 40 and 104 individuals with mean age ranging between 14.5 and 51.5 years. These studies reported number of female participants, which ranged between 19 and 57 individuals. The number of obese CP and nonobese CP patients ranged between 10 and 52 individuals, respectively. Two studies [16, 24] collected GCF only while other six studies [6, 17, 19, 23, 25, 26] collected both GCF and blood samples. Two studies [20, 21] collected GCF and tear fluid for the evaluation of cytokine levels. All studies [6, 16, 17, 19–26] employed commercial enzyme-linked immunosorbent assay (ELISA) for the detection of cytokine levels.

Four studies reported similar levels of IL-6 [6, 17, 19, 24] and five studies reported similar levels of adiponectin [6, 16, 17, 19, 24] between obese and nonobese individuals with CP, whereas 3 studies reported comparable levels of GCF leptin among CP patients with and without obesity [6, 19, 24]. One study [6] showed higher levels of TNF-*α* in obese CP subjects as compared to nonobese CP subjects. However, in four studies [16, 17, 19, 24], TNF-*α* was comparable among CP patients with and without obesity. Fadel et al. [24] showed similar levels of IL1-*β*, IL-8, and plasminogen activator inhibitor-1 (PAI-1) in obese and nonobese patients with CP. Modéer et al. [16] showed higher levels of IL-1*β* and IL-8 in obese CP patients and similar levels of PAI-1 in obese CP patients in comparison to nonobese patients with CP. Resistin concentration was found to be similar between obese and nonobese patients with CP in four studies [6, 19, 23, 24]. Two studies [25, 26] reported similar levels of CRP in periodontitis patients with and without obesity. Overall, a total of 8 studies [6, 16, 17, 19, 23–26] showed comparable cytokine levels among CP subjects with and without obesity, whereas a total of 5 studies [6, 16, 20, 25, 26] showed significantly raised cytokine levels in obese CP as compared to nonobese CP subjects (Table 2).

### 3.3. Quantitative Results of the Studies

#### 3.3.1. TNF-*α* and IL-6

**Figure 2:**
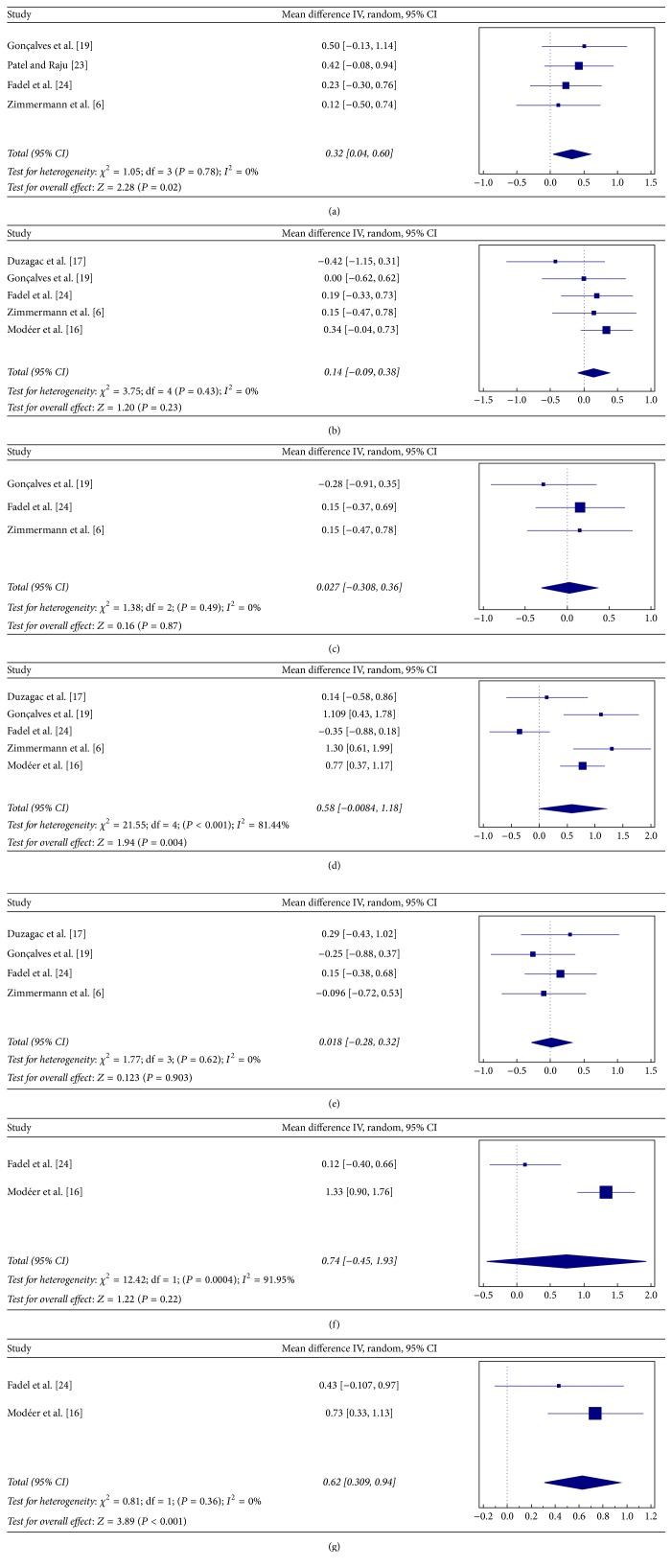
Forest plots presenting standard mean difference (SMD) of GCF cytokine levels between chronic periodontitis (CP) with and without obesity for (a) resistin; (b) adiponectin; (c) leptin; (d) TNF-*α*; (e) IL-6; (f) IL-8; and (g) IL-1*β*.

The overall mean difference in TNF-*α* levels between obese CP and nonobese CP patients was significant (SMD = 0.58; *Z* = 1.94; and *P* = 0.004; Figure 2(d)). The variability in differences in TNF-*α* levels was also significant (*Q*-value = 21.55; *P* < 0.001; and *I* ^2^ = 81.44%). IL-6 showed no significant difference in the GCF of obese CP and nonobese CP groups in all the four studies [6, 17, 19, 24] (SMD = 0.018; *Z* = 0.12; and *P* = 0.903; Figure 2(e)). The heterogeneity in levels of IL-6 between studies was also not significant (*Q*-value = 1.77; *P* = 0.62; and *I* ^2^ = 0%).

#### 3.3.2. Resistin, Adiponectin, and Leptin

Obese participants with CP showed significantly higher resistin levels than nonobese CP subjects (SMD = 0.32; *Z* = 2.28; and *P* = 0.02; Figure 2(a)). The heterogeneity in resistin between studies was not significant (*Q*-value = 1.05; *P* = 0.78; and *I* ^2^ = 0%). On the other hand, adiponectin levels showed no significant difference in both overall mean difference (SMD = 0.14; *Z* = 1.20; and *P* = 0.23; Figure 2(b)) and heterogeneity (*Q*-value = 3.75; *P* = 0.43; and *I* ^2^ = 0%) between the studies. These three studies [6, 19, 24] also showed no significant difference in leptin levels among obese and nonobese patients with CP, with mean difference (SMD = 0.027; *Z* = 0.16; and *P* = 0.87; Figure 2(c)) and heterogeneity being not significant (*Q*-value = 1.38; *P* = 0.49; and *I* ^2^ = 0%).

#### 3.3.3. IL-8 and IL1-*β*

The overall mean difference in IL-8 levels between obese and nonobese patients with CP showed no significant difference (SMD = 0.74; *Z* = 1.22; and *P* = 0.22; Figure 2(f)). The heterogeneity in GCF levels of IL-8 between studies was however significant (*Q*-value = 12.42; *P* = 0.0004; and *I* ^2^ = 91.95%). Obese participants with CP were found to have significantly higher GCF levels of IL1-*β* than nonobese CP (SMD = 0.628; *Z* = 3.895; and *P* < 0.001; Figure 2(g)). However, the heterogeneity for IL1-*β* between the studies was not significant (*Q*-value = 0.81; *P* = 0.36; and *I* ^2^ = 0%).

## 4. Discussion

The present systematic review assessed the GCF cytokine profile in CP patients with and without obesity. Eight studies [6, 16, 17, 19, 23–26] reported similar levels of cytokine (resistin, adiponectin, leptin, IL-6, IL-8, IL-10, IL1*β*, TNF-*α*, CRP, and PAI-1) among CP patients with and without obesity, while 5 studies [6, 16, 20, 25, 26] showed significantly higher levels of cytokine (IL-8, IL-1*β*, TNF-*α*, progranulin, MCP-4, and lipocalin) in obese CP patients as compared to nonobese CP subjects. Similarly, quantitative analysis showed IL-8, IL-1*β*, TNF-*α*, and resistin to be significantly higher in obese CP patients; however, adiponectin, leptin, and IL-6 were found comparable among obese and nonobese CP subjects. In periodontal inflammation, immune cells such as macrophages, leukocytes, and fibroblasts produce proinflammatory cytokines such as matrix metalloproteinases (MMPs), IL-1*β*, and receptor activator of NF-*κ*B ligand (RANKL) in response to bacterial challenge. These mediators play an essential role in extracellular matrix degradation and osteoclast differentiation and activation, therefore leading to collagen and bone destruction [30–32].

The premise of increased proinflammatory cytokines in obesity is such that the metabolic cells such as adipocytes initiate inflammation by triggering inflammatory signalling pathways [33]. This mediates a modest, low-level induction of inflammatory cytokines such as TNF-*α*, IL-1*β*, and IL-6 which occurs in response to excess nutrients. Overtime, this low-grade inflammation may induce the infiltration and activation of immune cells which is characterized by increase in the number of macrophages, mast cells, and T-lymphocytes that results in proinflammatory changes in the tissue environment and the inflammatory pathways and proinflammatory cytokines continue to reinforce. The inflammatory state therefore becomes maintained (chronic) and unresolved [33].

There are several explanations which can be posed regarding the similarity in cytokine levels in the GCF of CP patients with and without obesity. The studies [6, 16, 17, 19–26], which were included in this systematic review, were conducted with an aim to assess the level of cytokines in GCF as risk indicator for periodontal inflammation in obese patients. In the studies included [6, 16, 17, 19–26], the depth of periodontal pockets from which GCF was collected was not standardized among obese and nonobese patients. For instance, collection of GCF sampled sites from PD ≥ 5 mm was reported in some studies [6, 19, 22], whereas other studies did not report probing PD of GCF sampled sites [16, 17, 23–25] (Table 1). This may characterize a bias as level of periodontal inflammation in diseased periodontal pockets is known to influence GCF cytokine levels [34, 35]. Therefore it is hypothesized that the severity of localized periodontal inflammation on GCF cytokine levels in nonobese subjects could have exceeded the impact of obesity on GCF cytokine levels in obese CP patients. Moreover, in nearly half of the studies, the exclusion of patients with systemic diseases was not reported [6, 16, 23, 24]. It may therefore be speculated that the similarity in GCF cytokine profiles in obese and nonobese patients with CP could be associated with covert systemic conditions (such as diabetes) in the otherwise systemically healthy individuals [36, 37]. It may also be proposed that the balance between proinflammatory and anti-inflammatory mediators in periodontal tissues of nonobese subjects may be shifted towards a hyperinflammatory state that could impair the host response against pathogens and periodontal deterioration [38].

It is reported that leptin (expressed from adipocytes) shows an inverse relation with periodontal inflammation demonstrating a protective role in periodontal disease [39–41]. Interestingly, in the present review, GCF leptin levels were found comparable among obese and nonobese subjects [6, 19, 24]. A possible explanation for this may be derived from the fact that leptins release is stimulated by TNF-*α*, which is increased in CP patients as compared to healthy periodontium [42]. As all subjects included (obese and nonobese) in the studies reviewed had CP, the comparable stimulatory effect of TNF-*α* on leptin could have resulted in its similar levels in obese and nonobese subjects with CP. Therefore it may be hypothesized that periodontal inflammation may have a greater influence on GCF cytokine levels (including leptin) rather than the increased systemic inflammatory burden due to obesity.

It is well recognized that tobacco smoking has been shown to be deleterious for periodontal health [43, 44]. Studies on GCF assay have also shown cytokine concentrations to be low in habitual tobacco smokers owing to its immunosuppressant state as compared to nonsmokers [45]. Worthy of note, however, is that the subjects included in the studies fulfilling our inclusion criteria were nonsmokers. This again suggests that the intensity of periodontal inflammation alone may mainly be responsible for the increased GCF cytokine concentrations in subjects with periodontitis with and without obesity [46]. Weight management in obese subjects has shown a reduction in systemic inflammatory burden as expressed by lower levels of serum cytokines [47, 48]. It is also reported that weight control could reduce the amounts of MMP-8, MMP-9, and IL-1*β* in GCF of obese subjects with healthy periodontium [49]. The effect of periodontal therapy on obesity has been reported in recent study [50]; however, the effect of weight control on GCF cytokine profile in obese patients with CP still needs to be assessed. Moreover, the effect of periodontal treatment including recent adjunctive therapies (such as laser and photodynamic therapy) on the levels of proinflammatory needs to be explored [51, 52].

The review of included studies suggests that considerable heterogeneity existed in the studies reviewed (methodology, cytokines assessed, systemic health of subjects, and cytokine collection sites). Therefore, in light of the systematic review and assessment of available data, it remains arguable whether patients having chronic periodontitis with obesity have elevated proinflammatory GCF cytokine levels compared to nonobese individuals.

## 5. Conclusion

The present review suggests that the level of localized periodontal inflammation may have a greater influence on the GCF proinflammatory biomarker levels as compared to systemic obesity. Whether patients having chronic periodontitis with obesity have elevated proinflammatory GCF biomarkers levels compared to nonobese individuals remains debatable.

## Figures and Tables

**Table 1:** General characteristics of included studies.

Author et al., year	Study design; setting; country	Number of patients	Mean age/age range in years	Gender (M/F)	Periodontitis diagnostic criteria	GCF sample site	Sample characteristics: [sample type; collection tool; and storage temperature]
Duzagac et al. [17], 2015	Prospective non-RCT; university clinic; Turkey	45	Group 1: 40.66 (28–52) Group 2: 41.06 (28–51) Group 3: 39.66 (30–52)	19/26	PD ≥ 4 mm in ≥30% sites, BOP in ≥50% of sites, CAL > 2 mm in ≥20% sites, and radiographic evidence of bone loss	NA	GCF; paper strips; −80°C

Gonçalves et al. [19], 2015	Prospective non-RCT; university clinic; Brazil	40	Group 1: 50.0 (±4.5) Group 2: 48.5 (±9.3)	21/19	PD and CAL ≥ 4 mm in >30% teeth	Two noncontiguous deep sites (PD and CAL ≥ 5 mm with BoP)	GCF; paper strips; −80°C

Pradeep et al. [20], 2015	Cross-sectional; university clinic; India	40	Group 1: 32.5 (25–40) Group 2: 30.9 (25–40) Group 3: 31.0 (25–40) Group 4: 31.4 (25–40)	20/20	PD ≥ 5 mm, GI > 1, and CAL ≥ 3 mm with clinical signs of inflammation	NA	GCF; paper strips; −70°C

Pradeep et al. [21], 2016	Cross-sectional; university clinic; India	40	Group 1: 33.8 (±3.9) Group 2: 35.1 (±3.9) Group 3: 35.6 (±3.7) Group 4: 34.3 (±4.06)	20/20	PD ≥ 5 mm, GI > 1, and CAL ≥ 3 mm with clinical signs of inflammation	NA	GCF; paper strips; −70°C

Öngöz Dede et al. [22], 2016	Prospective non-RCT; university clinic; Turkey	90	Group 1: 47.13 (34–60) Group 2: 38.47 (26–51) Group 3: 35.73 (25–55) Group 4: 31.53 (25–53) Group 5: 41.33 (25–50) Group 6: 29.60 (25–33)	43/47	GI ≥ 2, PD and CAL ≥ 5 mm, and bone loss affecting >30% teeth	≥5 mm CAL, ≥6 mm PD, and ≥30% bone loss	GCF; paper strips; −40°C

Patel and Raju [23], 2014	Cross-sectional; university clinic; India	90	Group 1: NA (23–54) Group 2: NA (23–54) Group 3: NA (23–54)	45/45	GI > 1, PD ≥ 5 mm, CAL ≥ 3 mm, and evidence of radiographic bone loss	CAL ≥ 3 mm	GCF; micropipettes; 1 *μ*L; −70°C

Fadel et al. [24], 2014	Cross-sectional; obesity clinic; Sweden	55	Group 1: 15.0 (±1.0) Group 2: 16.0 (±2.0)	29/26	NA	NA	GCF; paper strips; −80°C

Zimmermann et al. [6], 2013	Cross-sectional; University clinic; Brazil	78	Group 1: 51.5 (±7.6) Group 2: 47.8 (±7.7) Group 3: 43.2 (±7.4) Group 4: 42.9 (±7.2)	21/57	≥30% sites with PD and CAL ≥ 4 mm and ≥4 noncontagious teeth with ≥1 site with PD and CAL ≥ 5 mm	Two noncontiguous deep sites (PD and CAL ≥ 5 mm with BoP)	GCF; paper strips; 4 *μ*L; −80°C

Pradeep et al. [25], 2013	Cross-sectional; university clinic; India	40	Group 1: 31.6 (25–45) Group 2: 32.8 (25–45) Group 3: 33.2 (25–45) Group 4: 31.4 (25–45)	20/20	PD ≥ 5 mm, GI > 1, and CAL ≥ 3 mm with clinical signs of inflammation	NA	GCF; paper strips; −70°C

Pradeep et al. [26], 2012	Cross-sectional; university clinic; India	40	Group 1: 36.8 (25–45) Group 2: 35.2 (25–45) Group 3: 35.2 (25–45) Group 4: 32.4 (25–45)	20/20	PD ≥ 5 mm, GI > 1, and CAL ≥ 3 mm with clinical signs of inflammation	NA	GCF; paper strips; −70°C

Modéer et al. [16], 2011	Cross-sectional; university clinic; Sweden	104	Group 1: 14.5 (11.0–17.9) Group 2: 14.5 (10.9–17.1)	58/46	≥1 sites with PD > 4 mm and alveolar bone loss ≥ 2 mm	NA	GCF; paper strips; −70°C

RCT: randomized clinical trial, M/F: male to female ratio, GCF: gingival crevicular fluid, PD: pocket depth, CAL: clinical attachment loss, BoP: bleeding on probing, GI: gingival index, and NA: not available.

**Table 2:** Cytokine profile in the crevicular fluid among study groups.

Author et al., year	Study groups	Type of assay	Cytokines evaluated	Main outcomes
Duzagac et al. [17], 2015	Group 1: OBCP (*n* = 15) Group 2: NBCP (*n* = 15) Group 3: NBNP (*n* = 15)	ELISA	Adiponectin, IL-6, TNF-*α*, and IL-10	GCF concentrations of adiponectin, IL-6, TNF-*α*, and IL-10 were comparable in OBCP and NBCP

Gonçalves et al. [19], 2015	Group 1: OBCP (*n* = 20) Group 2: NBCP (*n* = 20)	ELISA	Resistin, adiponectin, leptin, TNF-*α*, and IL-6	GCF concentrations of adiponectin, leptin, IL-6, TNF-*α*, and resistin were comparable in OBCP and NBCP

Pradeep et al. [20], 2015	Group 1: OBCP (*n* = 10) Group 2: NBCP (*n* = 10) Group 3: OBNP (*n* = 10) Group 4: NBNP (*n* = 10)	ELISA	Lipocalin-2	GCF lipocalin-2 concentrations were higher in OBCP compared to NBCP

Pradeep et al. [21], 2016	Group 1: OBCP (*n* = 10) Group 2: NBCP (*n* = 10) Group 3: OBNP (*n* = 10) Group 4: NBNP (*n* = 10)	ELISA	Vaspin	GCF vaspin levels were comparable in OBCP and NBCP

Öngöz Dede et al. [22], 2016	Group 1: OBCP (*n* = 15) Group 2: NBCP (*n* = 15) Group 3: OBG (*n* = 15) Group 4: NBG (*n* = 15) Group 5: OBNP (*n* = 15) Group 6: NBNP (*n* = 15)	ELISA	8-OHdG	GCF 8-OHdG levels were comparable in OBCP and NBCP

Patel and Raju [23], 2014	Group 1: OBCP (*n* = 30) Group 2: NBCP (*n* = 30) Group 3: NBNP (*n* = 30)	ELISA	Resistin	GCF resistin levels were comparable in OBCP and NBCP

Fadel et al. [24], 2014	Group 1: OBCP (*n* = 27) Group 2: NBCP (*n* = 28)	ELISA	IL-1*β*, IL-6, IL-8, TNF-*α*, leptin, resistin, PAI-1, adiponectin, and adipsin	GCF levels of IL-1*β*, IL-6, IL-8, TNF-*α*, leptin, resistin, PAI-1, adiponectin, and adipsin were comparable in OBCP and NBCP

Zimmermann et al. [6], 2013	Group 1: OBCP (*n* = 20) Group 2: NBCP (*n* = 20) Group 3: OBNP (*n* = 18) Group 4: NBNP (*n* = 20)	ELISA	Resistin, adiponectin, leptin, TNF-*α*, and IL-6	GCF concentrations of resistin, adiponectin, leptin, and IL-6 were comparable in OBCP and NBCP TNF-*α* level was higher in OBCP compared to NBCP

Pradeep et al. [25], 2013	Group 1: OBCP (*n* = 10) Group 2: NBCP (*n* = 10) Group 3: OBNP (*n* = 10) Group 4: NBNP (*n* = 10)	ELISA	MCP-4, hsCRP	GCF levels of MCP-4 were higher in OBCP as compared to NBCP GCF levels of hsCRP were comparable between OBCP and NBCP

Pradeep et al. [26], 2012	Group 1: OBCP (*n* = 10) Group 2: NBCP (*n* = 10) Group 3: OBNP (*n* = 10) Group 4: NBNP (*n* = 10)	ELISA	Progranulin, hsCRP	GCF levels of PGRN were higher in OBCP compared to NBCP GCF levels of hsCRP were comparable between OBCP and NBCP

Modéer et al. [16], 2011	Group 1: OBCP (*n* = 52) Group 2: NBCP (*n* = 52)	ELISA	Adiponectin, PAI-1, IL-1*β*, IL-8, and TNF-*α*	IL-1*β* and IL-8 were higher in OBCP compared to NBCP PAI-1, TNF-*α*, and adiponectin were comparable in OBCP and NBCP

OBCP: obese with periodontitis, NBCP: nonobese with periodontitis, OBNP: obese with no periodontitis; OBG: obese with gingivitis; NBG: nonobese with gingivitis, NBNP: nonobese with no periodontitis, GCF: gingival crevicular fluid, ELISA: enzyme-linked immunosorbent assay, TNF-*α*: tumor necrosis factor-alpha, IL: interleukin, hsCRP: high sensitivity c-reactive protein, 8-OhdG: 8-hydroxy-deoxyguanosine, ICAM: intercellular adhesion molecule, PAI-1: plasminogen activator inhibitor-1, and MCP-4: monocyte chemoattractant protein-4.

**Table 3:** Quality assessment using Newcastle-Ottawa scale of the included studies.

Investigators	Selection	Comparability	Exposure	Total score
Duzagac et al. [17]	**☆☆☆☆**	**☆**	**☆☆☆**	8
Gonçalves et al. [19]	**☆☆☆☆**	**☆☆**	**☆☆**	8
Pradeep et al. [20]	**☆☆☆**	**☆☆**	**☆☆**	7
Pradeep et al. [21]	**☆☆☆**	**☆☆**	**☆☆**	7
Öngöz Dede et al. [22]	**☆☆☆**		**☆☆☆**	6
Patel and Raju [23]	**☆☆☆☆**	**☆**	**☆**	6
Fadel et al. [24]	**☆☆☆**	**☆☆**	**☆**	6
Zimmermann et al. [6]	**☆☆☆☆**	**☆☆**	**☆**	7
Pradeep et al. [25]	**☆☆☆**	**☆☆**	**☆☆**	7
Pradeep et al. [26]	**☆☆☆**	**☆☆**	**☆☆**	7
Modéer et al. [16]	**☆☆☆**	**☆**	**☆☆**	6

## Appendix

### List of Excluded Studies: Reason for Exclusion Is Shown in Parenthesis

- Kâ K, Rousseau MC, Lambert M et al. Metabolic syndrome and gingival inflammation in Caucasian children with a family history of obesity. J Clin Periodontol. 2013; 40(11): 986–993 [cytokines studied in children].
- Kâ K, M. C. Rousseau, Tran SD et al. Circulating undercarboxylated osteocalcin and gingival crevicular fluid tumour necrosis factor-*α* in children. J Clin Periodontol. 2014; 41(5): 467–472 [cytokines studied in children].
- Buduneli N, Bıyıkoğlu B, Ilgenli T, Buduneli E, Nalbantsoy A, Saraç F, and Kinane DF. Is obesity a possible modifier of periodontal disease as a chronic inflammatory process? A case–control study. J Periodontal Res. 2014; 49(4): 465–471 [cytokines studied in serum].
- Fell RA, Zee KY, and Arora M. The correlation of serum and gingival crevicular fluid cytokines in obese subjects. J Int Acad Periodontol. 2013; 15: 20–28 [no control group].
- Lundin M, Yucel-Lindberg T, Dahllöf G, Marcus C, and Modéer T. Correlation between TNFa in gingival crevicular fluid and body mass index in obese subjects. Acta Odontol Scand. 2004; 62(5): 273–277 [no control group].
- Mendoza-Azpur G, Castro C, Peña L, Guerrero ME, De La Rosa M, Mendes C et al. Adiponectin, leptin and TNF-*α* serum levels in obese and normal weight Peruvian adults with and without chronic periodontitis. J Clin Exp Dent. 2015; 7(3): e380 [cytokines studied in serum].
- Nascimento GG, Leite FR, Correa MB, Peres MA, and Demarco FF. Does periodontal treatment have an effect on clinical and immunological parameters of periodontal disease in obese subjects? A systematic review and meta-analysis. Clin Oral Investig. 2015: 1–9 [review].
- Offenbacher S, Beck JD, Moss K, Mendoza L, Paquette DW, Barrow DA et al. Results from the Periodontitis and Vascular Events (PAVE) Study: a pilot multicentered, randomized, controlled trial to study effects of periodontal therapy in a secondary prevention model of cardiovascular disease. J Periodontol. 2009; 80(2): 190–201 [focus question not answered].
- Zhang L, Meng S, Tu Q, Yu L, Tang Y, Dard MM et al. Adiponectin ameliorates experimental periodontitis in diet-induced obesity mice. PloS One 2014; 9(5): e97824. doi: 10.1371/journal.pone.0097824 [experimental].
- Zuza EP, Barroso EM, Carrareto AL, Pires JR, Carlos IZ, Theodoro LH et al. The role of obesity as a modifying factor in patients undergoing non-surgical periodontal therapy. J Periodontol. 2011; 82(5): 676–682 [cytokines studied in serum].
